# Serum C-reactive protein level and sleep characteristics in obstructive sleep apnea syndrome comorbid with panic disorder: a preliminary study

**DOI:** 10.1186/s12888-023-05376-6

**Published:** 2023-11-20

**Authors:** Shuai Yang, Xiaoyi Kong, Xueyan Li, Yi-Jun Ge

**Affiliations:** 1 Department of Neurocritical Care Unit, Division of Life Sciences and Medicine, The First Affiliated Hospital of USTC, University of Science and Technology of China, Hefei, P. R. China; 2 Department of Neurology (Sleep Disorders), Affiliated Chaohu Hospital of Anhui Medical University, Hefei (Chaohu), P. R. China

**Keywords:** Obstructive sleep apnea syndrome, Panic disorder, Polysomnography

## Abstract

**Objective:**

Investigate the sleep characteristics of patients with obstructive sleep apnea syndrome (OSAS) comorbidity with panic disorder (PD), exploring its potential association with serum C-reactive protein (CRP) levels.

**Patients and methods:**

Fifty-four patients (25 OSAS patients with PD and 29 without PD) and 25 healthy controls (HCs) were included. The Self-rating anxiety scale (SAS), self-rating depression scale (SDS), and Pittsburgh sleep quality index (PSQI) were used to assess the mood and sleep quality of the subjects. All patients had circulating CRP levels and polysomnography was performed.

**Results:**

OSAS with PD had higher SAS, SDS, PSQI than the OSAS without PD. Compared to OSAS without PD, OSAS with PD had higher percentage of non- rapid eye movement sleep 1 and 2 (N1 and N2%), sleep latency, and a lower percentage of rapid eye movement sleep (REM%). Respiratory-related microarousal index, AHI, and time below 90% oxygen saturation (T90) were low, and the lowest oxygen saturation (LO2) was high. Serum CRP levels in OSAS patients with PD were lower than that in OSAS patients without PD, but higher than that in HCs. In OSAS patients with PD, serum CRP levels were negatively correlated with wake time after sleep onset and SAS scores but positively correlated with sleep efficiency and N2%. Serum CRP levels were positively correlated with T90 and negatively correlated with LO2.

**Conclusion:**

OSAS patients with PD had worse sleep quality, less severe OSAS, and low serum CRP levels. Serum CRP levels in OSAS patients with PD were associated with poorer sleep quality and duration of hypoxia rather than AHI.

## Introduction

Obstructive sleep apnea–hypopnea syndrome (OSAS) is a common sleep-disordered breathing disorder, with an estimated 425 million adults suffering from moderate-to-severe OSAS worldwide [1]. OSAS is characterized by recurrent closing or narrowing of the upper airway, resulting in intermittent hypoxia, sleep fragmentation, and recurrent nocturnal awakenings [2]. OSAS is a heterogeneous syndrome characterized by various pathophysiological mechanisms, clinical manifestations, and consequences of respiratory events [3]. Notably, the efficacy of OSAS treatments may also differ due to these characteristics. Therefore, identifying specific comorbidities can aid in developing more targeted diagnostic and therapeutic strategies [4].

Studies have found that OSAS is closely related to endocrine and cardiovascular diseases and can induce mood disorders [5]. Untreated OSAS may lead to the emergence of depressive and anxious emotions in patients, while prolonged emotional disorders can promote the progression of OSAS. Lee observed that more than half of the patients with OSAS have accompanying emotional problems [6]. Panic disorder (PD) is an extreme manifestation of anxiety disorders. Symptoms of nocturnal repetitive panic attacks in PD are similar to those of repetitive respiratory pauses in a patient with OSAS [7]. OSAS combined with PD patients often results in misdiagnosis or missed diagnoses. However, few studies have examined the relationship between OSAS and PD [8].

A follow-up study involving 8,704 OSAS patients found that the risk ratio for PD was 2.17 times higher in patients with sleep apnea [8]. In addition, a randomized crossover trial found that continuous positive airway pressure can not only reduce AHI, but also significantly reduce the number of panic attacks in OSAS patients with PD [9]. This indicates a close link between obstructive sleep apnea syndrome and PD. OSAS patients with PD may present with frequent arousal, racing heart, and breathlessness at night. Sleep complaints in OSAS patients with PD often depend on self-observed measures. In contrast, objective measures, including polysomnography, have not been observed in the literature on OSAS patients with PD.

Emerging evidence indicates that inflammation plays a significant role in the pathophysiology of OSAS and PD [10, 11]. Li et al. measured serum CRP levels in 156 patients with OSA and 110 healthy controls. Univariate logistic regression analysis showed that CRP levels were associated with the presence and severity of obstructive sleep apnea syndrome, and that the serum CRP levels of patients with severe OSA were significantly higher than those of patients with moderate OSA, suggesting that CRP may be part of the pathological mechanism of OSAS [12]. A recent meta-analysis found similar results [13]. Some studies have also found that CRP levels are not related to the degree of upper airway obstruction in OSA patients [11]. In addition, although several studies have shown that there is no difference in CRP levels between patients with PD and healthy controls, a meta-analysis involving 152 PD patients and 156 controls showed that the CRP levels of PD patients were significantly higher than those of the control group [14, 15]. There is also increasing evidence that immune-mediated inflammation is involved in the pathophysiological processes of psychiatric disorders. Investigating the levels of inflammation in the comorbid state of OSAS and PD may shed light on the critical pathophysiological connections between these two disorders.

This study investigated differences in sleep structure, the severity of respiratory events, and serum CRP levels between OSAS patients with and without PD. Furthermore, we explored the association between changes in serum CRP levels, poor sleep quality, and respiratory disturbances in OSAS patients with PD, thus providing the basis for clinical diagnosis and treatment.

## Methods

### Subjects

The patients came from the Department of Sleep Disorder, the Affiliated Chaohu Hospital of Anhui Medical University (China). The patients were diagnosed with OSAS following the Chinese Adult Obstructive Sleep Apnea Diagnosis and Treatment Guidelines [16]. Twenty-five individuals with PD according to the Diagnostic and Statistical Manual of Mental Disorders, Fifth Edition (DSM-5) criteria were enrolled [17]. The following conditions need to be met: recurrent unexpected panic attacks. After an attack, 1 or 2 of the following symptoms occurred and lasted for more than 1 month: a. Constantly worried or worried about the recurrence of a panic attack or its consequences. b. There were significant adverse changes in the behavior associated with panic attacks. This disorder cannot be attributed to a substance or drug. This disorder cannot be better explained by other mental disorders. The Structured Clinical Interview for DSM-5 Disorders, Clinical Version (SCID-5-CV) was administered to verify the diagnosis [18]. The exclusion criteria were if the patient (1) suffered from severe somatic diseases (including cardiovascular diseases, diabetes, hypertension, liver and kidney dysfunction, and infections, etc.), (2) met other diagnostic criteria for mental disorders according to the DSM-5, (3) had received systematic OSAS treatment (including positional changes, weight loss, and the use of a breathing machine, etc.), (4) was pregnant or lactating, and (5) exclude nocturnal panic attacks. A total of 25 healthy controls(HCs) undergoing physical examination in our hospital during the same period were included as controls: (a) without any mental disorders according to the DSM-5, (b) without somatic disease(including cardiovascular diseases, diabetes, hypertension, liver and kidney dysfunction, and infections, etc.), (c) without complaints related to sleep disorders, and (d) exclude pregnant or lactating women.

Liver and renal function tests, blood glucose, blood routine, urine routine, electrolytes, chest X-ray, and electrocardiogram were performed for all participants to exclude inflammation, infection or other organic diseases. This study was approved by the Ethics Committees of the Affiliated Chaohu Hospital of Anhui Medical University (Number KYXM-202108–005). All subjects signed a written informed consent form.

### General data collection

The general demographics of the patients were collected, including sex, age, and body mass index (BMI). Subjective sleep quality was assessed using The Pittsburgh Sleep Quality Index (PSQI). The scale includes seven dimensions: sleep onset time, sleep duration, sleep efficiency, sleep disturbance, subjective sleep quality, hypnotic drugs and daytime dysfunction. The threshold for sleep disturbance is 7. Higher total scores indicate poorer sleep quality [19]. The anxiety and depression levels of the participants were assessed using the Self-Rating Anxiety Scale (SAS) and Self-Rating Depression Scale (SDS) [20, 21]. The SDS scale consists of 20 items, and according to the Chinese standard, scores between 53 and 62 indicate mild depression, scores between 63 and 71 indicate moderate depression, and scores of 72 or higher indicate severe depression. The SAS scale also consists of 20 items, and according to the Chinese norm, the cutoff values for SAS standard scores are 50, with scores between 50 and 59 indicating mild anxiety, scores between 60 and 69 indicating moderate anxiety, and scores of 70 or higher indicating severe anxiety. The SAS and SDS scales have been widely used in China, and their reliability and validity have been evaluated in multiple studies [22, 23]. Furthermore, the assessment has been standardized for the Chinese population [24, 25].

### C-reactive protein

Serum CRP levels were measured in fasting venous blood in the morning using a fully automated biochemical analyzer (Siemens, Germany) in the morning after polysomnography was completed. All subjects had their blood samples collected at 7 o'clock in the morning.

### Polysomnography

All participants underwent one night of polysomnography (PSG) in the hospital's sleep monitoring to acclimatize to the novel environment, followed by two nights of PSG to collect the study data. The recording assessed sleep continuity and structure [26]. The former was measured as total sleep time (TST), wake time after sleep onset (WASO), sleep efficiency (SE), sleep onset latency (SL), percentages of stage 1, stage 2, stage 3, and REM sleep (N1, N2, N3, and REM%). Respiratory parameters, including apnea hypopnea index (AHI), time below 90% oxygen saturation (T90), and lowest oxygen saturation (LO_2_), respiratory event-related arousals (RA), REM sleep-related respiratory event-related arousals (REM-RA), NREM-related respiratory event-related arousals (NREM-RA), and average heart rate (HR) were recorded.

### Sample sizes

OSAS and healthy human serum CRP levels are based on the study of Bhushan et al. There are few studies on patients with OSAS combined with PD [27]. We calculated the average serum CRP level of the five initially enrolled patients. Sample size was estimated using GPower 3.1 Assuming a statistical power of 95%, significance level of 0.05, and effected difference of 1.07, it was estimated that at least 18 participants would be needed to show a statistically significant difference in among three groups [28].

### Statistical analysis

All data were analyzed using SPSS®20.0 for Windows (IBM Corp., Armonk, NY, USA). Normally distributed data are expressed as mean ± standard deviation (SD). Mann–Whitney U–tests were performed to analyze non-parametric data expressed as the 25^th^, 50^th^, and 75^th^ percentiles (P25, P50, and P75, respectively). The Kruskal–Wallis H-test was used for three group (HC, OSAS with and without PD) comparisons by adjusting the significance level to *P* < 0.0167 (Bonferroni correcting: 0.05/3). Spearman correlation was used to investigate relationships between the quantitative variables. Two-tailed *P* values ≤ 0.05 were considered statistically significant.

## Results

### General data and CRP levels of healthy controls and OSAS patients with and without PD

**Fig. 1:**
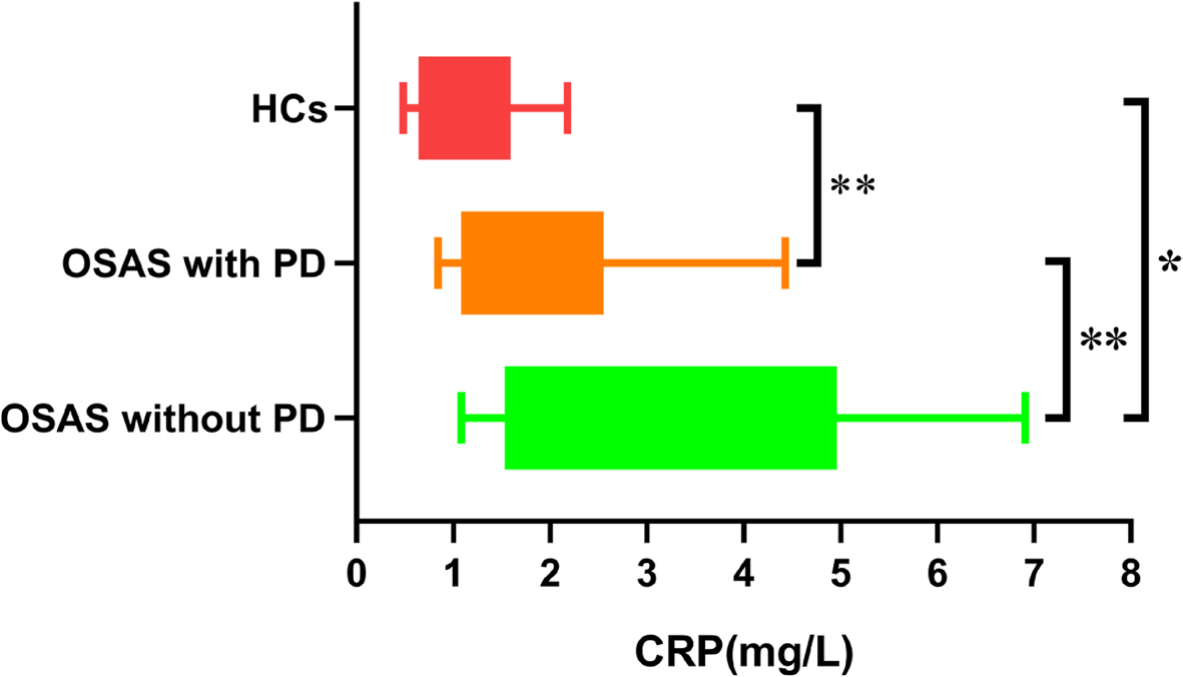
Comparison of serum CRP levels between OSAS patients with PD, without PD and healthy controls. ** *P* < 0.001; *P < 0.05. Abbreviations: OSAS, obstructive sleep apnea syndrome; PD, panic disorder; CRP, C-reactive protein

There were no significant differences in sex, age, or BMI between the three groups (Ps > 0.05). There was statistical significance in differences in SAS, SDS, and PSQI scores among the three groups. Compared to the HCs and patients without PD, patients with PD had significantly higher SAS, SDS, and PSQI scores (Ps < 0.001). Meanwhile, there were no significant differences in SAS, SDS, and PSQI scores between the OSAS patients without PD and HCs (Table 1). Serum CRP levels in OSAS patients with PD were lower than that in OSAS patients without PD (*P* < 0.001)., but higher than that in HCs (*P* < 0.05). (Fig. 1).

**Table 1:** General data of healthy controls and OSAS patients with and without PD

Variables	OSAS with PD (*n* = 25)	OSAS without PD (*n* = 29)	HC (*n* = 25)
Age	50.8 ± 12.5	46.6 ± 12.9	46.5 ± 12.2
Gender (male/female)	13/12	16/13	14/11
BMI (kg/m2)	26.8(24.5, 30.9)	28.3(26.2,30.8)	27.7(24.2, 30.7)
SAS score	63.0(53.8,65.7)^a,b^	33.0(27.3,37.8)	32.5(26.9,37.5)
SDS score	46.3(40.3,56.3)^a,b^	32.5(31.0,39.7)	32.5(31.3,35.0)
PSQI score	11.0(7.0,13.0)^a,b^	3.0 (2.5,6.0)	4.0(2.0,6.0)

*Abbreviations*: *BMI* Body mass index, *OSAS* Obstructive sleep apnea syndrome, *PD* Panic disorder, *SAS* Self-rating anxiety scale, *SDS* Self-rating depression scale, *PSQI* Pittsburgh sleep quality index

^a^indicates significance of the comparisons between healthy controls and OSAS patients

^b^indicates significance of the comparisons between OSAS with PD and without PD groups

### Sleep parameters of healthy controls and OSAS patients with and without PD

There was a statistical significance in differences in TST, WASO, SE, N1%, N2%, N3%, and REM% among the three groups. Compared to the HCs group, OSAS patients with PD had significantly higher WASO, SL, N1%, and significantly lower TST, SE, N3% and REM%. Patients with OSAS with PD had significantly higher SL, N1%, N2%, and significantly lower REM% than the OSAS without PD group (Table 2).

**Table 2:** Sleep parameters of healthy controls and OSAS patients with and without PD

Variables	OSAS with PD (*n* = 25)	OSAS without PD (*n* = 29)	HC (*n* = 25)
TST (min)	409.0 (284.8, 444.8)^a^	422.5 (366.0,485.5)	437.0 (424.8,467.0)
WASO (min)	55.8 (31.5, 91.1)^a^	81.0 (46.3,110.8)^a^	24.5 (18.6,33.0)
SL (min)	20.0 (11.5,36.3)^a**,**b^	9.0 (5.8,13.8)	6.8 (3.8,11.0)
SE	84.1% (72.2%, 87.6%)^a^	82.6% (76.5%,88.1%)^a^	91.5% (86.9%,94.2%)
N1%	39.7% (28.9%, 56.3%)^a**,**b^	27.0% (12.6%, 37.2%)^a^	10.4% (8.1%, 14.6%)
N2%	45.8% (35.9%, 57.0%)^a**,**b^	56.1% (42.9%, 70.3%)	54.0% (49.4%, 59.4%)
N3%	1.3% (0.0%, 5.2%)^a^	0.0% (0.0%, 5.4%)^a^	15.2% (11.7%, 20.5%)
REM%	7.9% (2.7%, 13.3%)^a**,**b^	14.4% (8.2%, 17.1%)^a^	18.3% (13.3%, 20.3%)

*Abbreviations*: *OSAS* Obstructive sleep apnea syndrome, *PD* Panic disorder, *TST* Total sleep time, *WASO* Wake time after sleep onset, *SE* Sleep efficiency, *SL* Sleep onset latency, *N1, N2, N3, and REM%* percentages of stage 1, stage 2, stage 3, and REM sleep

^a^indicates significance of the comparisons between healthy controls and OSAS patients

^b^indicates significance of the comparisons between OSAS with PD and without PD groups

### Respiratory parameters of healthy controls and OSAS patients with and without PD

There were statistically significant differences in AHI, LO2, T90, REM-RA, NREM-RA, RA, and HR among the three groups. OSAS patients with PD had significantly higher AHI, T90, REM-RA, NREM-RA, RA, and HR and significantly lower LO2 compared to HCs group. OSAS patients with PD had significantly lower AHI, T90, and REM-RA and higher LO2 than OSAS patients without PD (Table 3).

**Table 3:** Respiratory parameters of healthy controls and OSAS patients with and without PD

Variables	OSAS with PD (*n* = 25)	OSAS without PD (*n* = 29)	HC (*n* = 25)
AHI	9.2 (5.9, 21.0)^a,b^	44.3 (32.8, 56.3)^a^	0.0 (0.0, 0.0)
LO_2_	86.0 (83.0, 89.0)^a,b^	67.0 (59.5, 78.5)^a^	97.0 (97.0, 98.0)
T90(min)	1.15 (0.54, 10.11)^a,b^	43.59 (17.37, 148.30)^a^	0.0 (0.0, 0.0)
REM-RA	5.5 (0.0, 11.1)^a,b^	10.5 (6.4, 23.1)^a^	0.0 (0.0, 0.0)
NREM-RA	8.8 (5.8, 19.1)^a^	16.2 (6.8, 23.9)^a^	0.0 (0.0, 0.0)
RA	8.9 (5.6, 20.0)^a^	15.4 (6.2, 23.2)^a^	0.0 (0.0, 0.0)
HR	64.5 (58.0, 72.5)^a^	66.0 (63.0, 69.5)^a^	58.0 (56.0, 62.5)

*Abbreviations*: *OSAS* Obstructive sleep apnea syndrome, *PD* Panic disorder, *AHI* Arousal index, *T90* time with blood oxygen saturation < 90%, *LO2* the lowest SpO2, *REM-RA*, REM sleep-related respiratory event-related arousals, *NREM-RA* NREM-related respiratory event-related arousals, *RA* Respiratory event-related arousals, *HR* average heart rate. REM

^a^ indicates significance of the comparisons between healthy controls and OSAS patients

^b^ indicates significance of the comparisons between OSAS with PD and without PD groups

### Correlations among serum CRP, sleep parameters, and respiratory parameters in OSAS patients with PD

**Fig. 2:**
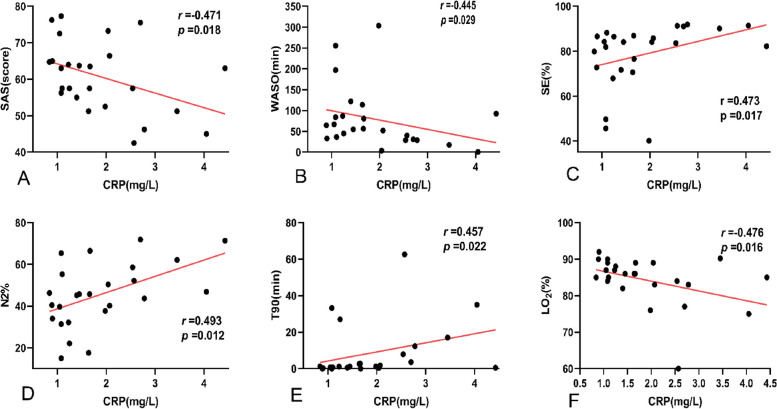
Correlation analysis revealed significant associations between CRP and sleep parameters, and respiratory parameters in OSAS patients with PD: **A** SAS score (*r* =  − 0.471, *P* < 0.05), **B** WASO (*r* =  − 0.445, *P* < 0.05), **C** SE (*r* = 0.473, *P* < 0.05), **D** N2% (*r* = 0.493, *P* < 0.05), **E** T90 (*r* = 0.457, *P* < 0.05), **F** LO2 (*r* =  − 0.476, *P* < 0.05). Abbreviations: SAS, self-rating anxiety scale; WASO, wake time after sleep onset; SE, sleep efficiency; N2%, percentages of stage 2; T90, time with blood oxygen saturation < 90%; LO2, the lowest SpO2

In OSAS patients with PD, serum CRP levels were negatively correlated with WASO (*r* =  − 0.445, *P* < 0.05) and SAS score (*r* =  − 0.471, *P* < 0.05) but positively correlated with SE (*r* = 0.473, *P* < 0.05) and N2% (*r* = 0.493, *P* < 0.05). In terms of respiratory parameters, serum CRP levels were positively correlated with T90 (*r* = 0.457, *P* < 0.05) and negatively correlated with LO2 (*r* =  − 0.476, *P* < 0.05) in OSAS patients with PD. However, there was no correlation between serum CRP levels and AHI (*P*** > **0.05) in the OSAS patients with PD (Fig. 2).

## Discussion

OSAS is a systemic disease with multiple organ involvement. It is considered an independent risk factor for the development of cardiovascular diseases [29]. However, some studies have ignored the significant correlation between OSAS and psychiatric disorder. PD and OSAS are both well-known and common diseases. Therefore, it can challenge the co-occurrence of two diseases in clinical decision making. Panic attacks can be mistaken for arousal caused by nocturnal hypoxemia and upper airway collapse in patients with OSAS, thus delaying treatment [30]. Still, using anxiolytics to control panic attacks is dangerous before excluding OSAS by PSG in patients with PD. This is because anxiolytics can aggravate airway collapse, exacerbating OSAS [31]. Therefore, identifying patients with coexisting OSAS and PDs would help optimize treatment and diagnosis for patients with OSAS with PD [32].

The current study represents the first to evaluate sleep-breathing characteristics and CRP levels in OSAS patients with PD. Our study shows that OSAS patients with PD had worse sleep quality, lower AHI values, and lower serum CRP levels compared to OSAS patients without PD. Serum CRP levels were associated with poorer sleep quality and duration of hypoxia in OSAS patients with PD.

### OSAS patients with PD had more worse sleep quality and severe mood disorders

Currently, there are only a few studies about objective sleep architecture in OSAS patients with PD. All patients in this study group underwent PSG for two nights, which can objectively reflect the actual sleep state of patients at night, and has specific clinical value. Compared to patients without PD, OSAS patients with PD have increased sleep latency, light sleep, and decreased sleep time in the REM phase. Combined with the PSQI score, it is clear that patients with OSAS with PD have poor subjective sleep quality, and their objective sleep quality is significantly worse than that of patients with OSAS without PD.OSAS patients with PD have hypothalamic⁃pituitary⁃adrenocortical axis hyperfunction and increased alertness due to worry and anxiety, causing an increase in the duration/ratio of light sleep (i.e., increased duration/ratio of sleep in N1, N2, or both phases) [33]. Studies have shown that abnormal noradrenergic neurons in the brain are associated with the pathogenesis of PD and that neurological dysfunction of norepinephrine and 5-hydroxytryptamine leads to nocturnal panic attacks and central respiratory dysfunction [34, 35]. In turn, norepinephrinergic neurons in the brain are a critical factor in maintaining REM sleep, which may also contribute to reduced REM sleep in OSAS patients with PD [36]. Surprisingly, the PSG results showed a reduction in TST time in OSAS patients with PD compared to OSAS patients without PD. Still, there was no statistical difference between the two groups, which may be explained by the small sample size.

Additionally, anxiety symptoms were more pronounced in OSAS patients with PD, as seen in SAS scores. Anxiety also reduces sleep quality at night in OSAS patients with PD. Therefore, OSAS patients with PD had more severe anxiety and poorer sleep quality offset upper airway collapse, resulting in atypical nocturnal OSAS symptoms in OSAS patients with PD. This leads to their apnea events being masked in the initial stages of the disease, thus ignoring OSAS. Apnea hypoventilation events in OSAS patients with PD are of concern only when they develop further and worsen. However, OSAS may have caused severe damage to the organism at this time. Therefore, PSG should be perfected in patients with PD, especially those with significant nocturnal seizures, to prevent underdiagnosis of OSAS.

### OSAS patients with PD had had milder OSAS symptoms

Regarding respiratory parameters, OSAS patients without PD have increased AHI due to recurrent complete or partial upper airway obstruction during sleep, apnea, and hypoventilation, resulting in recurrent hypoxia. However, both apnea and hypoxemia were less severe in the OSAS patients with PD than in the OSAS patients without PD, suggesting a lesser degree of apnea and hypoxic events in OSAS patients with PD. This may be due to the lower arousal threshold in patients with PD [37]. Although the relationship between OSAS and upper airway obstruction was previously believed to be primarily related to anatomical factors, in recent years, the role of non-anatomical factors has received increasing attention from researchers [38]. Arousal is one of the most studied non-anatomical factors [39]. During obstructive breathing, patients gradually increase their inspiratory effort. When it exceeds the threshold for arousal, the brain wakes up from sleep, which can reopen the airway, restore oxygen saturation, and relieve hypercapnia in patients with OSAS [40]. The merger of panic disorder results in a decrease in the arousal threshold and an increase in vigilance in patients. This makes them more prone to awakening from pathological events such as airway obstruction, leading to a shorter duration of hypoxia in patients [41]. On the other hand, previous studies have suggested that an essential mechanism for the occurrence of panic symptoms is increased sensitivity to carbon dioxide (CO2) in patients [42]. High sensitivity may cause patients with comorbid PD to overreact to minor changes in CO2, leading to the early termination of respiratory events [43].

This study also found that the respiratory-related microarousal index was higher in the REM phase in OSAS patients with PD compared to OSAS patients without PD. This may be due to a more significant decrease in mandibular electromyographic activity in the REM phase compared to the awake and NREM phases, more severe apnea, and more respiratory-related arousals [44, 45]. Unexpectedly, although sleep disturbances were more severe in the OSAS patients with PD than in the OSAS patients without PD, there were no significant differences between the OSAS patients with PD and without PD in the total number of respiratory-related microarousals and the mean nocturnal heart rate.

### Lower inflammation levels in OSAS patients with PD are associated with sleep and OSAS symptoms

The present study is the first to explore CRP serum concentrations in OSAS patients with PD. The level of CRP, which is synthesized in the liver, is a commonly used inflammatory biomarker [46]. According to recent meta-analyses, OSAS patients without PD had higher serum CRP levels than those in the control group [47]. Li also found that patients with severe OSA had significantly higher serum CRP levels than those with moderate OSAS [12]. In our study, although serum CRP levels were higher for OSAS patients with PD than for healthy people, they were lower for OSAS patients without PD. This may be because OSAS severity is milder in OSAS patients with PD. Our study also found that serum CRP levels in OSAS patients with PD were positively correlated with LO_2_and negatively correlated with T90. This indicates that the decrease in inflammation levels is related to the reduction of hypoxia symptoms in OSAS patients without PD. As mentioned above, PD may be reduce hypoxic events by lowering the threshold of awakening of the patient [48]. Patients with combined PD exhibit milder hypoxia, which reduces inflammation levels. Contrary to our expectations, serum CRP levels in OSAS patients without PD were not correlated with AHI. This suggests that alterations in OSAS-related inflammation may be mainly due to hypoxemia rather than AHI-induced changes.

Regarding limitations, first, as a cross-sectional study, this research cannot identify cause-and-effect relationships between OSAS and PD. There is a close association between OSAS and PD, but the specific mechanism remains unclear. Also, this study is a single-center study with a small overall sample size, so its results need to be further confirmed by large-scale clinical studies. Although we have recognized the impact of nocturnal panic attacks on sleep architecture, it is important to note that this study did not analyze such patients as a specific subgroup.

At present, there are few studies on OSAS patients with PD. Furthermore, the coexistence of these two conditions often leads to misdiagnosis and underdiagnosis. When encountering patients with complaints such as nocturnal snoring and awakening from sleep, physicians in both internal medicine in general hospitals tend to primarily consider a diagnosis of OSAS, neglecting the evaluation of the patients' psychiatric symptoms. Similarly, psychiatrists who come across patients with recurrent nocturnal awakenings accompanied by symptoms such as palpitations and sweating often think of panic disorder, thereby overlooking the occurrence of OSAS. Therefore, clinicians should pay attention to improving polysomnography and mood assessment to determine the possibility of comorbidity and achieve rational diagnosis and treatment.

## Conclusion

In conclusion, this study revealed that patients suffering from OSAS combined with PD demonstrate notably diminished sleep quality compared to OSAS without PD. The excessive worry in patients with combined PD leads to increased light sleep and decreased arousal threshold. They are more prone to awaken from airway obstruction, resulting in shorter duration of hypoxia compared to the pure OSAS group. Additionally, we have found lower levels of CRP in OSAS patients with PD, which is associated with the duration of hypoxia rather than AHI. This study provides valuable insights into the understanding of the relationship between PD and OSAS and may serve as a basis for future research on potential therapeutic interventions.

## Data Availability

The datasets are available from the corresponding author (geyijun1984@126.com) at any request.

## Abbreviations

OSAS: Obstructive sleep apnea syndrome
PD: PD
TST: Total sleep time
WASO: Wake time after sleep onset
SE: Sleep efficiency
SL: Sleep onset latency
N1, N2, N3, and REM%: Percentages of stage 1, stage 2, stage 3, and REM sleep; arousal index, T90, time with blood oxygen saturation < 90%
LO2: The lowest SpO2
REM-RA: REM sleep-related respiratory event-related arousals
NREM-RA: NREM-related respiratory event-related arousals
RA: Respiratory event-related arousals
HR: Average heart rate
